# The Use of Probiotic *Megasphaera elsdenii* as a Pre-Harvest Intervention to Reduce *Salmonella* in Finishing Beef Cattle: An In Vitro Model

**DOI:** 10.3390/microorganisms10071400

**Published:** 2022-07-12

**Authors:** Kellen Habib, James Drouillard, Vanessa de Aguiar Veloso, Grace Huynh, Valentina Trinetta, Sara E. Gragg

**Affiliations:** Department of Animal Sciences and Industry, Kansas State University, 1530 Mid-Campus Drive North, Manhattan, KS 66506, USA; kellenhabib@ksu.edu (K.H.); jdrouill@ksu.edu (J.D.); veloso@vet.k-state.edu (V.d.A.V.); gphuynh@ksu.edu (G.H.); vtrinetta@ksu.edu (V.T.)

**Keywords:** *Megasphaera elsdenii*, *Salmonella*, cattle, ruminal fluid, feces, in vitro

## Abstract

Reducing *Salmonella* in cattle may mitigate the risk of transmission through the food chain. *Megasphaera elsdenii* (ME) is a microorganism found naturally in the bovine rumen that can be administered as a probiotic to mitigate ruminal acidosis. Understanding the impact of feeding ME to *Salmonella* populations in cattle was the objective of this study. Bovine ruminal fluid (RF) and feces were inoculated with antibiotic susceptible or resistant *Salmonella* and treated with varying concentrations of ME. *Salmonella* was enumerated at 0, 24, 48, and 72 h using the most probable number (MPN). Volatile fatty acids (VFAs) and pH were recorded from non-inoculated samples. Treating RF with ME did not significantly impact *Salmonella* concentration or VFA production (*p* > 0.05). The pH of RF and feces decreased over time (*p* ≤ 0.05). *Salmonella* concentration declined in feces, with the largest reduction of 1.92 log MPN/g and 1.05 log MPN/g observed for antibiotic susceptible *Salmonella* between 0 and 72 h by the 2.5 × 10^5^ CFU/g and control (0.0 CFU/g) concentration of ME, respectively. Treating RF with ME did not impact *Salmonella* concentration. *Salmonella* concentration in feces decreased, although ME must be further investigated before a conclusion regarding efficacy in vitro can be determined.

## 1. Introduction

Throughout production, cattle are exposed to a variety of microorganisms in the feedlot environment. Antibiotics historically have been administered sub-therapeutically as growth promotants to optimize performance and increase profits. In January of 2017, the Food and Drug Administration’s ban on the use of “medically important” antibiotics for growth promotion became effective [1,2]. This, coupled with public health implications associated with a rise in antibiotic resistance [1] among pathogenic microorganisms, emphasizes the need for novel management practices to control microbial populations without introducing selective pressure associated with “medically important” antibiotics.

Understanding the impact of alternative animal husbandry practices to control foodborne pathogens, such as *Salmonella*, in cattle should also be prioritized. As Acuff and Dickson summarize in their recent book [3], the relationship between these pathogens and cattle is well-documented. This is further complicated by the fact that cattle often show no clinical symptoms of infection while shedding *Salmonella* in their feces [4,5,6] and/or harboring *Salmonella* in their lymph nodes [7]. In a 2014 review paper, Wells et al. [8] reviewed the body of research seeking to understand relationships between pathogens and other factors, such as diet and microbiome. Although these and other factors represent complex relationships within the host, it is clear that diet can impact cattle’s pathogen shedding status.

Supplementing cattle with a direct-fed microbial (DFM) has been investigated as a pre-harvest intervention to control foodborne pathogens while also improving production efficiency [9,10,11]. *Megasphaera elsdenii* is a lactic acid-utilizing bacterium that plays an important role in the rumen microbiome [12,13]. This microorganism utilizes lactate and mitigates ruminal acidosis (RA), which can occur as a result of feeding cattle diets containing relatively large proportions of cereal grains and other concentrates. Lactate utilization by *M. elsdenii* results in the production of VFAs (butyrate and acetate from d-lactate) [14] and moderation of ruminal pH [13]. Although *M. elsdenii* has demonstrated value as a mitigant for RA, published literature is lacking regarding the impact of this probiotic on *Salmonella* shedding in cattle. Previous publications suggest that an increase in fecal VFAs results in decreased *E. coli* O157:H7 populations in the hindgut of cattle [15,16]. Recognizing that *M. elsdenii* produces VFAs from lactate in the rumen, it could be hypothesized that *M. elsdenii* may also be present and producing VFAs in the hindgut, thereby reducing *Salmonella* carriage in the rumen and entire gastrointestinal tract, resulting in a reduction in fecal shedding.

The objective of this study is to use an in vitro challenge model previously developed by our team [17] to determine the efficacy of *M. elsdenii* at reducing antibiotic susceptible and resistant *Salmonella* populations in cattle feces and ruminal fluid. The data generated may provide preliminary information to support a larger in vivo cattle feeding trial.

## 2. Materials and Methods

### 2.1. Experimental Design

An in vitro challenge model described by Page et al. [17] was utilized to determine if *M. elsdenii* is effective at reducing *Salmonella* populations in the rumen and feces of cattle. This study was completed as a completely randomized design. Antibiotic susceptible and resistant *Salmonella* serotypes were inoculated into ruminal fluid and feces, with or without *M. elsdenii*, and quantified throughout a 72 h period. All procedures were replicated three times and efficacy was determined based upon *Salmonella* reductions achieved by *M. esldenii* treated samples in comparison to the inoculated, untreated control. The pH and concentration of volatile fatty acids were also measured throughout the study from non-inoculated samples.

### 2.2. Culture Preparation

A two-strain *Salmonella* cocktail was prepared from one *Salmonella* Newport strain and one *Salmonella* Anatum strain, both of which were pansusceptible to the following veterinary antibiotics: amoxicillin/clavulanic acid, ampicillin, cefoxitin, ceftiofur, ceftriaxone, chloramphenicol, ciprofloxacin, gentamicin, nalidixic acid, streptomycin, sulfisoxazole, tetracycline, and trimethoprim/sulfamethoxazole. A second two-strain cocktail was prepared from one *Salmonella* Newport and one *Salmonella* Montevideo, both of which were resistant to one or more of the veterinary antibiotics listed above. Frozen stock cultures of each strain were streaked for isolation onto Xylose Lysine Desoxycholate (XLD) agar and incubated for 18–24 h at 37 °C. Following incubation, one isolated colony was selected from each plate, transferred to a 9 mL tryptic soy broth (TSB) tube, and incubated for 18–24 h at 37 °C. Following incubation, 1 mL of each strain was diluted in 0.1% peptone water (PW) to prepare separate antibiotic susceptible and resistant cocktails, each with an approximate concentration of 1.0 × 10^6^ CFU/mL.

### 2.3. Sample Preparation

Ruminal fluid and feces were collected from cattle at the Kansas State University Beef Cattle Research Center according to Institutional Animal Care and Use Committee approvals. Feces were collected from multiple animals and composited into one large fecal sample. The same procedure was followed for ruminal fluid. From the fecal and ruminal fluid composite samples, a 500 g portion was weighed for inoculation (target concentration of 1.0 × 10^3^ CFU/g to 1.0 × 10^4^ CFU/g). Briefly, 1 mL of cocktail was homogenized by shaking the ruminal fluid or hand massaging feces for 2 min. The inoculated 500 g was divided into 90 g portions (*n* = 5 for ruminal fluid and feces) into individual Whirl-Pak^®^ (Nasco, Madison, WI, USA) sample bags and *M. elsdenii* was added using Lactipro NXT^®^ (MS Biotec, Wamego, KS, USA), a commercially available freeze-dried *M. elsdenii* product that is commonly administered to feedlot cattle as an oral drench and has a guaranteed concentration of 5.0 × 10^8^ CFU/mL when rehydrated in the provided anaerobic diluent. Lactipro NXT^®^ was rehydrated using the anaerobic diluent provided with the Lactipro NXT^®^ product, based upon the 5.0 × 10^8^ CFU/mL guaranteed concentration, to achieve target concentrations of *M. elsdenii* in each sample. Briefly, from each prepared concentration, 10 mL were added to 90 g of ruminal fluid and feces (90 g of ruminal fluid or feces + 10 mL of *M. elsdenii* = 100 g of sample total) to prepare samples containing *M. elsdenii* at concentrations described in Table 1.

Thieszen et al. [18] investigated ruminal pH and VFA production in cattle orally drenched with 0, 25, 50, 75, and 100 mL of Lactipro NXT^®^ (previously known as Lactipro) per animal. The concentrations in Table 1 were selected to reflect similar concentrations to Thieszen et al. [18], with the addition of a 5.0 × 10^8^ CFU/g concentration that would simulate the growth of *M. elsdenii* in the rumen following Lactipro NXT^®^ administration. Recognizing that the rumen typically holds a minimum of 95 L (~25 gallons) [19], a ruminal volume of 100 L was used to calculate target concentrations for *M. elsdenii* to maintain the same concentration in each 100 g sample of ruminal fluid and feces (Table 1). These procedures were completed for each cocktail.

A set of ruminal fluid and fecal samples not inoculated with *Salmonella* (hereafter to referred to as non-inoculated) was also prepared according to Table 1 for *Salmonella* MPN (as previously described), as well as VFA and pH analyses as described in the Volatile Fatty Acid and pH Analyses section below. Therefore, the result of this sample preparation was three separate experiments for ruminal fluid and feces as follows: non-inoculated feces, non-inoculated ruminal fluid, feces inoculated with antibiotic-susceptible *Salmonella*, ruminal fluid inoculated with antibiotic-susceptible *Salmonella*, feces inoculated with antibiotic-resistant *Salmonella*, ruminal fluid inoculated with antibiotic-resistant *Salmonella*.

Following preparation of samples, individual sample bags were placed in anaerobic containers with an AnaeroGen (Oxoid, Lenexa, KS, USA) gas sachet and anaerobic indicator strip to simulate anaerobiosis of the bovine gastrointestinal tract. Anaerobic containers were stored on a shaking incubator set to 50 rpm and 38.6 °C to mimic peristaltic motion within the gastrointestinal tract and the body temperature of a bovine, respectively. Samples were removed from the shaking incubator and analyzed for *Salmonella* at 0, 24, 48, and 72 h using a repeated measures approach. Following sampling, the samples were returned to the anaerobic containers and incubator as described above.

### 2.4. Microbiological Analyses

Ruminal fluid and feces were enumerated via the most probable number (MPN) as described by Page et al. [17]. Briefly, at each time point, 10 g of each sample were homogenized in 90 mL of PW, serially diluted in 9 mL PW tubes, and used to inoculate a 3 × 7 matrix of Rappaport Vasiliadis (RV) broth. The RV MPN tubes were incubated for 18–24 h at 37 °C and then streaked onto Xylose Lysine Desoxycholate (XLD). The XLD plates were incubated for 18–24 h at 37 °C and examined for typical (black colonies) *Salmonella* growth. *Salmonella* growth on the XLD plate corresponded to a positive MPN tube. The presence or absence of *Salmonella* was recorded for each tube and the pattern of tubes was recorded for the 3 × 7 MPN matrix. The MPN CFU/g was calculated for each sample using the Environmental Protection Agency (EPA) MPN calculator [20]. Any RV tubes associated with questionable *Salmonella* growth on XLD were subjected to immunomagnetic separation (IMS) as described by Page et al. [17]. Briefly, anti-*Salmonella* Dynabeads (Applied Biosystems, Grand Island, NY, USA) were used with a KingFisher ML (Thermo Scientific, Waltham, MA, USA) according to manufacturer’s guidelines, and 50 µL of the resultant bead suspension were spread-plated onto XLD. The XLD plates were incubated and observed for characteristic *Salmonella* growth as previously described. Growth was associated with a positive RV tube and the MPN pattern was then finalized based upon these IMS data. *Salmonella* on XLD plates from non-inoculated and inoculated samples were agglutinated at random using Wellcolex^TM^ latex agglutination kits (Remel, Lenexa, KS, USA) to ensure that growth was being accurately reported.

### 2.5. Volatile Fatty Acid and pH Analyses

At each sampling point, the pH of each ruminal fluid and fecal sample was recorded using a calibrated benchtop pH meter with glass probe. Fecal samples were prepared for pH measurement by homogenizing 5 g with 20 mL of sterile water [21]. Following pH measurement, the homogenized fecal samples were centrifuged and supernatant collected for VFA analyses [21]. Concentrations of VFA were evaluated for each ruminal fluid and fecal sample at every time period. Briefly, concentrations of volatile fatty acids in ruminal fluid and feces were determined by gas chromatography using an Agilent 7890 gas chromatograph equipped with a flame ionization detector and bonded polyethylene glycol capillary column (DB-Wax Ul; 20 m length × 0.18 mm diameter × 18 µm film thickness; Agilent; Santa Clara, CA, USA). Hydrogen was used as the carrier gas and oven temperature was ramped from 50 to 240 °C at a rate of 30 °C/min.

### 2.6. Statistical Analyses

Three replications of the study were completed each for ruminal fluid and feces. Log MPN/g of *Salmonella* were analyzed as a repeated measures study using linear mixed models (MIXED procedure of Statistical Analysis Software; SAS 9.4, Cary, NC, USA) to determine impact of *M. elsdenii* on concentrations of *Salmonella* in cattle ruminal fluid and feces. Statistical analyses were performed separately for ruminal fluid and feces, as well as for non-inoculated, antibiotic-susceptible *Salmonella*, and antibiotic-resistant *Salmonella* samples (e.g., antibiotic-susceptible *Salmonella* inoculated in ruminal fluid was a single statistical model). Each sample bag served as the experimental unit. The best covariance structure was determined for each sample and inoculation type (e.g., ruminal fluid, non-inoculated), and then used in the model. Main effects of treatment, time, and the time × treatment interaction were evaluated at the 0.05 significance level. Means and standard error of the mean (SEM) were calculated for significant main effects and interactions using the LSMEANS statement with Tukey–Kramer to evaluate differences between means. Mixed-effects analyses with the Tukey–Kramer adjustment in GraphPad Prism 9.0 (San Diego, CA, USA) were used to analyze impact of *M. elsdenii* on VFAs and pH of non-inoculated samples. Main effects of treatment, time, and time × treatment interaction were evaluated at the 0.05 significance level.

## 3. Results

### 3.1. Microbiological Analyses

When feces were treated with varying concentrations of *Megasphaera elsdenii* and sampled at 0, 24, 48, and 72 h, the impact of treatment, time, and the treatment × time interaction was significant (*p* < 0.0001) for naturally occurring *Salmonella* in non-inoculated feces. Because the treatment × time interaction was significant, non-inoculated *Salmonella* data from feces are displayed and discussed according to sampling point (time) and *Megasphaera elsdenii* concentration (treatment). In general, the naturally occurring *Salmonella* concentration declined over time (Table 2) in non-inoculated feces. The largest reduction observed was 1 log MPN/g (*p* < 0.0001) between the 0 and 72 h time points in feces treated with 5.0 × 10^8^ CFU/g of *M. elsdenii*. However, naturally occurring *Salmonella* also declined (*p* < 0.0001) by 0.76 log MPN/g in the control feces (0.0 CFU/g of *M. elsdenii*) not inoculated with *Salmonella*. At the 72 h timepoint, naturally occurring *Salmonella* concentrations in non-inoculated feces ranged from 0.63 to 0.87 log_10_ MPN/g in feces treated varying concentrations of *Megasphaera elsdenii*, and all *Salmonella* concentrations were statistically the same (*p* > 0.05).

**Figure 1:**
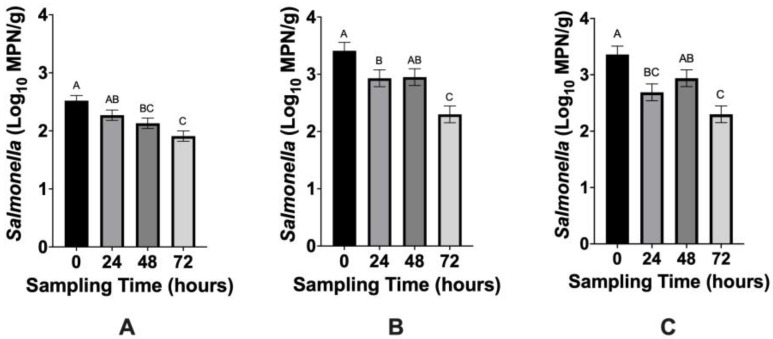
(**A**–**C**). Mean *Salmonella* concentration (log_10_ MPN/g) in cattle ruminal fluid that is (**A**) non-inoculated, (**B**) inoculated with pansusceptible *Salmonella*, (**C**) inoculated with *Salmonella* that is resistant to one or more antibiotics at 0, 24, 48, and 72 h following treatment with varying concentrations of *Megasphaera elsdenii* as an in vitro model. Error bars represent standard error of the mean. Sampling points with different superscripts are statistically different (*p* ≤ 0.05).

The impact of treatment (*p* = 0.0720) and the treatment × time interaction (*p* = 0.4641) were not apparent for ruminal fluid samples not inoculated with *Salmonella*, but time of incubation did affect *Salmonella* recoveries (*p* = 0.0001). Naturally occurring *Salmonella* declined in non-inoculated ruminal fluid from 2.52 log MPN/g at 0 h to 1.91 log MPN/g at 72 h (Figure 1A; *p* < 0.0001).

The main effects of time, treatment, and the treatment × time interaction significantly (*p* < 0.0001) impacted antibiotic-susceptible *Salmonella* concentrations in feces treated with varying concentrations of *Megasphaera elsdenii*. Because the treatment × time interaction was significant, antibiotic-susceptible *Salmonella* data from feces are displayed and discussed according to sampling point (time) and *Megasphaera elsdenii* concentration (treatment). The largest antibiotic-susceptible *Salmonella* reduction (1.92 log MPN/g) was achieved between 0 and 72 h by the 2.5 × 10^5^ CFU/g *M. elsdenii* concentration (Table 3; *p* < 0.0001). Antibiotic-susceptible *Salmonella* in the control feces (0.0 CFU/g of *M. elsdenii*) declined by 1.05 log MPN/g between the 0 and 72 h timepoints (*p* < 0.0001). At the 72 h timepoint, the largest population of antibiotic-susceptible *Salmonella* was recovered from control feces that were not treated with *M. elsdenii* (0.0 CFU/g of *M. elsdenii*), with 3.31 log MPN/g recovered in comparison to the 2.44 to 2.87 log MPN/g of *Salmonella* recovered from feces inoculated with varying concentrations of *M. elsdenii*.

Neither treatment (*p* = 0.7840) nor treatment × time interaction (*p* = 0.3746) impacted *Salmonella* counts for ruminal fluid samples inoculated with antibiotic-susceptible *Salmonella,* thus data are discussed only in accordance with the effect of time (*p* < 0.0001). Antibiotic-susceptible *Salmonella* populations in ruminal fluid declined from 3.41 log MPN/g at 0 h to 2.30 log MPN/g at 72 h (Figure 1B; *p* < 0.0001).

When sampling feces at 0, 24, 48, and 72 h following treatment with varying concentrations of *Megasphaera elsdenii*, the main effect of time (*p* < 0.001) and the treatment × time interaction (*p* = 0.0136) impacted *Salmonella* counts for feces inoculated with antibiotic-resistant *Salmonella*. Effects of *Megasphaera elsdenii* treatment were not evident (*p* = 0.4974). Because the treatment × time interaction was significant, antibiotic-resistant *Salmonella* data from feces are displayed and discussed according to the sampling point (time) and *Megasphaera elsdenii* concentration (treatment). The largest antibiotic-resistant *Salmonella* reduction (1.60 log MPN/g) was achieved between 0 and 72 h by the 2.5 × 10^5^ CFU/g *M. elsdenii* concentration (Table 4; *p* = 0.1283). Antibiotic-resistant *Salmonella* in the control feces (0.0 CFU/g of *M. elsdenii*) declined by 0.70 log MPN/g between the 0 and 72 h timepoints and this reduction was not significant (*p* = 0.7570). At the 72 h timepoint, the largest population of antibiotic-resistant *Salmonella* was recovered from control feces (0.0 CFU/g of *M. elsdenii*), with 3.30 log MPN/g recovered in comparison to the 2.43 to 2.90 log MPN/g of antibiotic-resistant *Salmonella* recovered from feces inoculated with varying concentrations of *M. elsdenii*. A large standard error was observed for samples collected at the 0 h sampling point, which impacted statistical differences when 0 h means were compared to other sampling points (Table 4).

The impact of treatment (*p* = 0.3813) and the treatment × time interaction (*p* = 0.4574) were not evident for ruminal fluid samples inoculated with antibiotic-resistant *Salmonella*. Time (*p* < 0.0001) was a significant main effect and data are only discussed according to the main effect of time. Antibiotic-resistant *Salmonella* concentrations in ruminal fluid declined from 3.36 log MPN/g at 0 h to 2.29 log MPN/g at 72 h (Figure 1C; *p* < 0.0001).

### 3.2. VFA and pH Analyses

**Figure 2:**
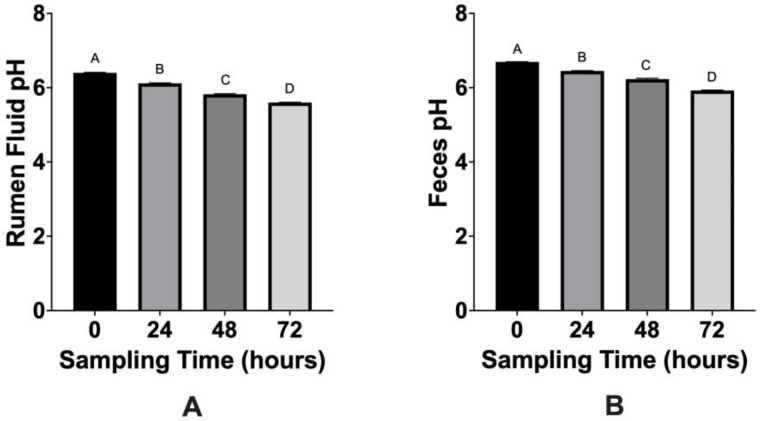
(**A**,**B**). Mean pH of cattle ruminal fluid (**A**) and cattle feces (**B**) at 0, 24, 48, and 72 h following treatment with varying concentrations of *Megasphaera elsdenii* as an in vitro model. Error bars represent standard error of the mean. Sampling points with different superscripts are statistically different (*p* ≤ 0.05).

The main effects of time, treatment, and the time × treatment interaction were not significant (*p* > 0.05) for acetate, propionate, isobutyrate, butyrate, isovalerate, valerate, isocaproate, caproate, and heptanoate in ruminal fluid and feces. The main effect of treatment, and the time × treatment interaction, did not significantly (*p* > 0.05) impact the pH of ruminal fluid or feces. The main effect of time did impact pH for ruminal fluid (*p* < 0.0001) or feces (*p* < 0.0001), with pH declining throughout the study. Ruminal fluid pH declined from 6.4 at 0 h to 5.6 at 72 h (Figure 2A; *p* < 0.0001) and fecal pH declined from 6.7 at 0 h to 5.9 at 72 h (Figure 2B; *p* < 0.0001).

## 4. Discussion

Supplementing finishing beef cattle diets with *M. elsdenii* has been shown to promote ruminal health [13,22] and improve dry matter intake [22]. Thus, from the perspective of animal performance and health, a benefit exists to supplementing finishing diets with this probiotic microorganism. However, the impact of *M. elsdenii* on *Salmonella* carriage in cattle has not been investigated. Therefore, this study was completed to address knowledge gaps surrounding efficacy of *M. elsdenii* as a pre-harvest intervention to reduce the burden of *Salmonella* in cattle.

In general, *Salmonella* populations declined in feces that were naturally or artificially contaminated. The pH of feces significantly declined over time (*p* ≤ 0.05) from 6.7 at 0 h to 5.9 at 72 h, which may have contributed to the decline in *Salmonella* populations. The largest reduction in antibiotic resistant and susceptible *Salmonella* populations was observed between 0 and 72 h, with a 1.92 log MPN/g (2.5 × 10^5^ CFU/g of *M. elsdenii*) reduction in antibiotic-susceptible *Salmonella* populations in inoculated feces. This reduction was 0.87 log MPN/g greater than the 1.05 log MPN/g reduction observed in the control feces inoculated with antibiotic-susceptible *Salmonella*. Similarly, at the 72 h timepoint, feces treated with *M. elsdenii* harbored fewer (*p* ≤ 0.05) antibiotic susceptible and resistant *Salmonella* populations than control feces, although the largest difference was 0.87 log MPN/g for both antibiotic susceptible and resistant *Salmonella* populations. Because these reductions are less than 1 log MPN/g, the efficacy of *M. elsdenii* at reducing antibiotic susceptible or resistant *Salmonella* in cattle feces should be considered marginal, as the biological relevance of reductions less than 1 log MPN/g is minimal, and additional research is necessary before efficacy can be determined.

The addition of *M. elsdenii* to feces did not significantly change VFA concentrations or pH (*p* > 0.05), although the pH of feces declined throughout the study (*p* ≤ 0.05). An increase in VFAs in feces has been associated with decreased populations of *E. coli* O157:H7 in the hindgut of cattle [15,16]. In the present study, *M. elsdenii* did not significantly alter VFA production, yet *Salmonella* populations generally declined throughout the study, with the largest reduction observed in feces treated with 2.5 × 10^5^ CFU/g of *M. elsdenii*. This suggests that 1) reductions were not associated with VFA concentration, or 2) given that only a 0.87 log MPN/g disparity was observed in fecal *Salmonella* populations at 72 h, the concentration of VFAs was consistently high enough in all samples to decrease *Salmonella* to a similar degree (within 1 log MPN/g difference). It is also important to note that pH and VFAs were determined from separate, non-inoculated fecal samples and may not fully represent the pH or VFA concentration of each inoculated sample.

The lack of a treatment effect suggests that *M. elsdenii* is not effective at reducing antibiotic susceptible or resistant *Salmonella* populations in cattle ruminal fluid when used as an intervention in an in vitro model. Although naturally occurring and inoculated *Salmonella* populations declined throughout the 72 h sampling period (Figure 1), these reductions were not associated with a treatment effect. The pH of ruminal fluid significantly declined over time (*p* ≤ 0.05) from 6.4 at 0 h to 5.6 at 72 h, which may have contributed to *Salmonella* population declines. Bolton et al. [23] also inoculated ruminal fluid with *Salmonella* and reported that changes in ruminal fluid pH (6.61 to 5.77) did not impact *Salmonella* populations, which suggests that pH changes in the present study may not have been responsible for reductions in *Salmonella*. It is also important to consider that cattle ruminal fluid consists of extensive native microbiota [17,24], which also provides competition for *Salmonella* and may impact survival. As mentioned previously, pH and VFAs were determined from separate, non-inoculated ruminal fluid samples and may not fully represent the pH or VFA concentration of each inoculated sample.

*Megasphaera elsdenii* is an important microorganism in the rumen of cattle [12,13] and is associated with VFA production [14]. However, the addition of *M. elsdenii* to ruminal fluid in the present study did not significantly change VFA concentrations or pH (*p* > 0.05). Bolton et al. [23] also discussed how the ruminal environment is relatively unfavorable for *Salmonella* due to VFAs; however, similar to the present study, also reported a reduction in *Salmonella* populations in the rumen that were not associated with VFA production.

## 5. Conclusions

The data presented herein provide preliminary evidence that *M. elsdenii* is not effective at reducing *Salmonella* in ruminal fluid but may be effective at marginally reducing *Salmonella* in cattle feces (<1 log MPN/g in comparison to control feces) when used in an in vitro model. The in vitro model presents challenges, however, including exposure to oxygen during sampling points, the lack of nutrient infusion (i.e., a bovine eating), and the accumulation of metabolic end-products during the 72 h study period. *M. elsdenii* is an anaerobic microorganism [14] and the viability may have declined due to exposure to oxygen during sample preparation and sampling periods when samples were not held under anaerobic conditions. The populations of *M. elsdenii* were not enumerated throughout the study due to challenges with selectivity and interference from the native microflora in ruminal fluid and feces. However, a method that can demonstrate *M. elsdenii* viability from even these complex matrices and a challenge model that does not require exposure to oxygen should be considered for future research. Ultimately, additional research is necessary, including in vivo feeding trials, before the efficacy of *M. elsdenii* at reducing the burden of *Salmonella* in cattle can be determined.

## Figures and Tables

**Table 1:** Addition of Lactipro NXT^®^ in 10 mL anaerobic diluent to achieve target concentrations of *Megasphaera eldenii* in cattle ruminal fluid and feces as an in vitro model.

Target Concentrations of *M. elsdenii* (CFU/g)
Control: 10 mL anaerobic diluent without *M. elsdenii* = 0
1.0 × 10^5^
2.5 × 10^5^
5.0 × 10^5^
5.0 × 10^8^

**Table 2:** Mean concentration (log_10_ MPN/g) of naturally occurring *Salmonella* in non-inoculated cattle feces at 0, 24, 48, and 72 h following treatment with varying concentrations of *Megasphaera elsdenii* as an in vitro model.

Time Point (Hours)	*Megasphaera elsdenii* Target Concentration (CFU/g)									
	0.0		1.0 × 10^5^		2.5 × 10^5^		5.0 × 10^5^		5.0 × 10^8^	
	*Salmonella* Log MPN/g	SEM	*Salmonella* Log MPN/g	SEM	*Salmonella* Log MPN/g	SEM	*Salmonella* Log MPN/g	SEM	*Salmonella* Log MPN/g	SEM
0	1.52 ^A,a^	0.01479	1.31 ^A,b^	0.01479	1.46 ^A,a,c^	0.01479	1.44 ^A,c^	0.01479	1.63 ^A,d^	0.01479
24	1.54 ^A,a^	0.01688	1.18 ^B,b^	0.01688	1.37 ^B,c^	0.01688	1.05 ^B,d^	0.01688	1.44 ^B,c^	0.01688
48	1.37 ^A,a^	0.09226	1.37 ^A,B,a^	0.09226	1.43 ^A,B,a^	0.09226	1.22 ^A,B,a^	0.09226	1.40 ^A,B,a^	0.09226
72	0.76 ^B,a^	0.0765	0.65 ^C,a^	0.0765	0.87 ^C,a^	0.0765	0.67 ^C,a^	0.0765	0.63 ^C,a^	0.0765

Uppercase superscripts that vary within a column are statistically different (*p* ≤ 0.05) and are comparing one treatment across each sampling point. Lowercase superscripts that vary within a row are statistically different (*p* ≤ 0.05) and are comparing each treatment across a single sampling point. SEM indicates standard error of the mean.

**Table 3:** Mean concentration (log_10_ MPN/g) of susceptible *Salmonella* concentration in inoculated cattle feces at 0, 24, 48, and 72 h following treatment with varying concentrations of *Megasphaera elsdenii* as an in vitro model.

Time Point (Hours)	*Megasphaera elsdenii* Target Concentration (CFU/g)									
	0.0		1.0 × 10^5^		2.5 × 10^5^		5.0 × 10^5^		5.0 × 10^8^	
	Log MPN/g	SEM	Log MPN/g	SEM	Log MPN/g	SEM	Log MPN/g	SEM	Log MPN/g	SEM
0	4.36 ^A,a^	0.06504	4.31 ^A,a^	0.06504	4.44 ^A,a^	0.06504	4.32 ^A,a^	0.06504	4.31 ^A,a^	0.06504
24	3.62 ^B,a,b^	0.02313	3.54 ^B,b^	0.02313	3.67 ^B,a^	0.02313	3.64 ^B,a^	0.02313	4.05 ^B,c^	0.02313
48	3.54 ^B,a^	0.03557	3.37 ^C,b^	0.03557	2.54 ^C,c^	0.03557	3.31 ^C,b,d^	0.03557	3.18 ^C,d^	0.03557
72	3.31 ^B,a^	0.1186	2.44 ^D,b^	0.1186	2.52 ^C,b^	0.1186	2.68 ^D,b^	0.1186	2.87 ^C,a,b^	0.1186

Uppercase superscripts that vary within a column are statistically different (*p* ≤ 0.05) and are comparing one treatment across each sampling point. Lowercase superscripts that vary within a row are statistically different (*p* ≤ 0.05) and are comparing each treatment across a single sampling point. SEM indicates standard error of the mean.

**Table 4:** Mean concentration (log_10_ MPN/g) of *Salmonella* that is resistant to one or more antibiotics in inoculated cattle feces at 0, 24, 48, and 72 h following treatment with varying concentrations of *Megasphaera elsdenii* as an in vitro model.

Time Point (Hours)	*Megasphaera elsdenii* Target Concentration (CFU/g)									
	0.0		1.0 × 10^5^		2.5 × 10^5^		5.0 × 10^5^		5.0 × 10^8^	
	Log MPN/g	SEM	Log MPN/g	SEM	Log MPN/g	SEM	Log MPN/g	SEM	Log MPN/g	SEM
0	3.97 ^A,a^	0.6618	4.00 ^A,B,a^	0.6618	4.10 ^A,a^	0.6618	3.93 ^A,B,a^	0.6618	3.57 ^A,a^	0.6618
24	3.67 ^A,a^	0.3022	3.70 ^A,a^	0.3022	3.27 ^A,a^	0.3022	3.23 ^A,B,a^	0.3022	3.80 ^A,a^	0.3022
48	3.53 ^A,a^	0.04092	3.40 ^A,a,b^	0.04092	2.50 ^A,d^	0.04092	3.30 ^A,b,c^	0.04092	3.20 ^A,c^	0.04092
72	3.30 ^A,a^	0.1287	2.43 ^B,b^	0.1287	2.50 ^A,b^	0.1287	2.67 ^B,b^	0.1287	2.90 ^A,a,b^	0.1287

Uppercase superscripts that vary within a column are statistically different (*p* ≤ 0.05) and are comparing one treatment across each sampling point. Lowercase superscripts that vary within a row are statistically different (*p* ≤ 0.05) and are comparing each treatment across a single sampling point. SEM indicates standard error of the mean.

## Data Availability

The data presented in this study are available upon request from the corresponding author.
